# The Influence of Fish Length on Tissue Mercury Dynamics: Implications for Natural Resource Management and Human Health Risk

**DOI:** 10.3390/ijerph10020638

**Published:** 2013-02-06

**Authors:** Dana K. Sackett, W. Gregory Cope, James A. Rice, D. Derek Aday

**Affiliations:** 1 Department of Oceanography, University of Hawaii, 1000 Pope Rd, Honolulu, HI 96822, USA; 2 Department of Environmental and Molecular Toxicology, North Carolina State University, Box 7633, Raleigh, NC 27695, USA; E-Mail: greg_cope@ncsu.edu; 3 Department of Biology, North Carolina State University, Box 7617, Raleigh, NC 27695, USA; E-Mails: jim_rice@ncsu.edu (J.A.R.); derek_aday@ncsu.edu (D.D.A)

**Keywords:** fish size, mercury, fishery regulation, advisory, length limit

## Abstract

Consumption of fish has well-known human health benefits, though some fish may contain elevated levels of mercury (Hg) that are especially harmful to developing children. Fish length is most often the basis for establishing fishery harvest regulations that determine which fish will ultimately be consumed by humans. It is, therefore, essential to quantify the relationship between fish length and Hg accumulation in regard to harvest regulations for effective fishery and public health policy. We examined this relationship for three sportfish from six lakes across North Carolina, USA. Bluegill (*Lepomis macrochirus*) had the lowest Hg levels and only the very largest fish in the most contaminated site exceeded the US Environmental Protection Agency (USEPA) Hg screening level. Black crappie (*Pomoxis nigromaculatus*) had an intermediate level of Hg and larger individuals exceeded the USEPA screening level; however, they tended not to exceed this level before reaching the harvest length limit. Largemouth bass (*Micropterus salmoides*) exceeded the USEPA screening level at sizes below the fishery length limit in two lakes, leaving only higher risk fish for anglers to harvest and consume. Removing the effects of fish age and trophic position, we found strong positive correlations between Hg and fish length for largemouth bass and black crappie. We suggest public health officials and wildlife managers collaborate to structure fishery regulations and length-based fish consumption advisories that protect consumers from Hg exposure and communicate the relative risk of fish consumption.

## 1. Introduction

Mercury (Hg) contamination of aquatic systems is a significant health concern for humans and wildlife, as evidenced by numerous consumption advisories for common fish species found throughout the United States (US) [1] and elsewhere in the world [2]. Human consumption of fish contaminated with methylmercury (MeHg), the highly neurotoxic and bioavailable form that accumulates in fish, dictates the need to protect consumers, particularly women of childbearing age and young children, by providing dietary advice and public health recommendations [3]. To accurately assess consumption risk and effectively communicate that risk to the public, it is essential to have a better understanding of mechanisms that affect Hg accumulation in fish tissue. Fish consumption is the primary vector for Hg movement from aquatic systems to people [4] and fish length is often the basis for establishing recreational and commercial harvest regulations, directly influencing the fish that are harvested for consumptive purposes. Given the central role that length plays in both understanding contaminant dynamics and in determining natural resource policy, it could be argued that the effect of fish length on Hg contamination is one of the most important relationships to understand, particularly for harvested species. Our concern is that fishery length limits (regulations specifying which sizes of fish are legal to keep) meant to protect the sustainability of the fishery often inadvertently limit anglers to harvesting only the largest, and therefore most contaminated, fish. In this study, we examined the relationship between tissue Hg and fish length in the context of fishery harvest regulations and public health.

Though we know a great deal about factors that are associated with fish tissue Hg [5,6,7], studies that link harvest regulations and contaminant dynamics are rare, and those that have directly quantified the relationship between Hg and fish length often simply report a positive correlation [8,9,10]. While some studies have quantified this relationship in more detail [11,12], uncertainty remains regarding factors (e.g., trophic position, age) that could confound interpretation of this relationship [13,14,15]. For example, many studies have attributed the positive relationship between tissue Hg and fish length to confounding or correlated effects of age and trophic position [8,16,17]. Fish age is typically positively correlated with fish length and fish tissue Hg due to slow elimination rates (half-life on the order of years) [18,19], leading to Hg accumulation in tissue as fish consume more food through time [8,20,21]. In addition, because fish are generally gape limited and often exhibit ontogenetic diet shifts [22], as fish get larger their trophic level typically increases as they consume larger (and, therefore, often more contaminated) prey [23,24]. One aim of our work was to disentangle these factors to determine whether fish length would drive tissue Hg independently of age and trophic position. Aside from understanding the effects of these confounding factors, it remains the case that there is a strong positive relationship between fish tissue Hg and body length [10,16,17], and body length is frequently the metric used in communication between public health policy makers and anglers. Thus, we focused much of the analysis in this study on the relationship between fish tissue Hg and fish length, particularly in relation to harvest regulations for public health protection.

It is also unclear whether the relationship between fish length and tissue Hg is constant or is a function of system-specific Hg contamination levels. Much Hg research is directed at understanding the differences in MeHg dynamics among aquatic systems because consumption of contaminated fish is the most direct path for Hg to affect the health of humans and wildlife, particularly given that nearly all (95–99%) of the Hg in fish tissue exists in the form of MeHg [25,26,27]. Several studies have linked environmental factors to rates at which Hg is methylated by bacteria in different systems. For example, acidic or low pH water has been identified as an important predictive factor for high fish tissue Hg in many studies [28,29,30]. Additionally, low-lying systems prone to flooding, such as swamps and wetlands, have been identified as areas likely to have greater concentrations of MeHg in fish tissue [31,32]. To improve contaminant risk assessment and the communication of relative risk to anglers, directly assessing the relationship between fish length and Hg accumulation among different sites with varying levels of Hg contamination, particularly in regard to fishery harvest regulations, is a necessary step.

The overall goal of this research was to directly quantify the relationship between fish body length and tissue Hg levels for three commonly harvested and consumed freshwater species, largemouth bass (*Micropterus salmoides*), black crappie (*Pomoxis nigromaculatus*), and bluegill (*Lepomis macrochirus*), in the context of fishery harvest regulations and public health protection. Specifically, we measured fish tissue Hg in these species across a range of lengths from systems with different levels of Hg contamination [30]. We identified length thresholds for each species above which human health risks from fish consumption presumably increase (based on North Carolina (NC), USEPA and US Food and Drug Administration (USFDA) Hg screening or action levels), and compared those to typical harvest length limits for each species. We also quantified how the relationship between fish tissue Hg and body length changed based on differences in levels of Hg contamination among lakes and examined this association in relation to two confounding factors, fish age and trophic position. Furthermore, it is often not feasible for state public health or resource management agencies to sample a wide size range of each fish species during routine sampling [33]. Therefore, we also determined whether mean fish tissue Hg obtained by the North Carolina Division of Water Quality (NCDWQ; as a proxy for other state agencies with similar missions) during sixteen years of routine sampling could be used to predict the length at which fish would reach a screening level in a system. 

## 2. Methods

### 2.1. Site Selection and Sampling

**Figure 1:**
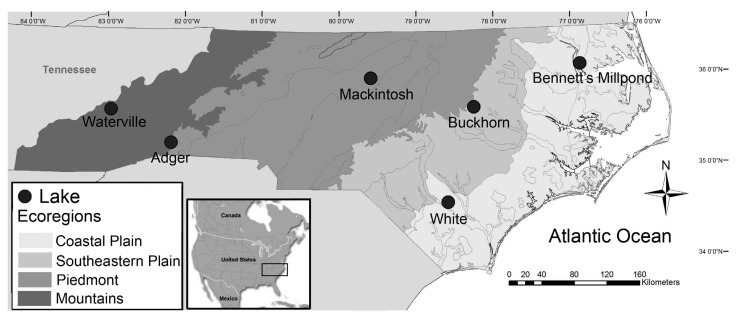
Locations of study lakes in North Carolina, USA, in relation to ecoregions as defined by Griffith *et al.* [42]; lakes were selected to represent a range of fish tissue Hg contamination based on a previous study [30].

We sampled six lakes in North Carolina with different levels of Hg contamination (Figure 1). At each lake, we attempted to collect 15 largemouth bass, 15 bluegill, and 15 black crappie with electrofishing: five each from “small” (bluegill <115; largemouth bass <355; black crappie <203 mm total length, TL), “medium” (115–150; 355–432; 203–279 mm TL) and “large” (>150; >432; >279 mm TL) size classes to ensure a wide range of body lengths were collected for each species. Power analysis in our previous research suggested that five individuals of a restricted length range would be sufficient to represent Hg concentrations in those species within the specified length range [34]. These length ranges were also chosen to ensure that five fish were collected below the typical fishery length limit (355 mm for largemouth bass and 203 mm for black crappie; bluegill typically do not have a length limit), five were within an intermediate size range for anglers to harvest and consume (115–150 mm for bluegill, 355–432 mm for largemouth bass, and 203–279 mm for black crappie), and five were large enough to be considered quality catches in most systems (>150 mm for bluegill, >432 mm for largemouth bass, and >279 mm for black crappie) [35]. The fishery length limits selected here are commonly used for North Carolina waterbodies, as well as waterbodies in other states [36,37,38]. We chose these particular species because they are widely distributed, often consumed by humans, and typically represent distinct trophic levels. Largemouth bass are common apex predators that feed primarily on fish but also on benthic invertebrates [39]. Black crappie are also piscivorous at larger sizes, feeding on small fishes and invertebrates [40]. Bluegill occupy a lower trophic level, foraging on a mix of zooplankton, insects, and plant material [41]. Unfortunately, we were unable to collect all of the targeted black crappie. Black crappie were collected in four of the six study lakes, and we were only able to collect all targeted individuals in one of the study systems. All fish were collected from 15 March to 12 April 2010 to limit potential seasonal variation. 

Collected fish were measured (nearest mm TL) placed in food-grade sealable bags, and stored frozen (−20 °C) until processing. In the laboratory, fish were thawed, weighed (nearest g), and processed for Hg analyses using a trace metal-free dissection technique [43]. Briefly, each fish was skinned and filleted with a clean stainless steel knife on fresh aluminum foil with fresh nitrile gloves. The left dorsal filet was removed, weighed, freeze dried and ground into a homogenous powder. Because MeHg constitutes more than 95% of total mercury in fish [25], we analyzed total Hg, which is much more cost effective than analyzing MeHg. Total Hg was measured using USEPA method 7473 [43]. Briefly, approximately 100 mg of dried and homogenized tissue was analyzed by direct solids decomposition-atomic absorption using a Milestone DMA-80 direct Hg analyzer. Each batch of 10 samples was accompanied by appropriate quality assurance protocols, including blanks, Hg-spiked samples, duplicates, and National Research Council of Canada certified reference material (CRM) to calibrate the instrument (TORT-2 Lobster hepatopancreas tissue) and to assess method accuracy (TORT-2, DORM-2 Dogfish muscle tissue, DOLT-2 Dogfish liver tissue). In addition, several study samples were fortified with CRM (Sandy loam 3) to assess the potential matrix impact on mercury recovery. All blanks, spikes and CRMs were within acceptable limits. Recoveries of CRM ranged from 90% to 114%, with an overall mean recovery of 99.2% (SD, 6.7%). Dry tissue Hg concentrations were converted back to wet weight using the measured percent moisture for each sample.

### 2.2. Fish Processing and Analyses

The relative trophic status of each fish was estimated using nitrogen isotope analysis [44]. Benthic invertebrates were collected from a composite of three sediment samples taken in the general area of fish collection in each system to establish a baseline nitrogen isotopic signature [44]. Benthic invertebrates were sieved from sediment samples taken at each lake with a petite ponar dredge, identified to subclass, order or family, and preserved in ethanol [45]. A similar composite of benthic invertebrates that included Chironomidae, Ceratopogonidae, Trichoptera, Ephemeroptera, and Oligochaeta, were used for each lake. Tricoptera without a case, which are often predacious, were not included in our samples. In the laboratory, dried invertebrates and freeze-dried fish tissue samples were analyzed for nitrogen isotopes by combustion (1,000 °C) followed by a reduction from oxidized nitrogen to atmospheric nitrogen (N_2_), whereas water was removed using magnesium perclorate. This procedure used a Carlo Erba NC2500 Elemental Analyzer coupled with an AS200 auto sampler (Thermo Scientific, Waltham, MA, USA) to introduce samples to a DeltaV Advantage Isotope Ratio Mass Spectrometer (Thermo Finnigan, Bremen, Germany). Trophic position of each fish was determined from the ratio of N^15^ to N^14^ (δ^15^N) using the following equation from Vander Zanden and Rasmussen [46]: fish trophic position = (fish δ^15^N – baseline δ^15^N) / 3.4‰ + 2, where the baseline δ^15^N was the invertebrate δ^15^N measured in each lake, 3.4‰ represents the nitrogen isotope fractionation constant [47], and “+ 2” accounts for the trophic level of the invertebrates used as the baseline [48].

Fish age was determined for each individual via otolith analysis. Otoliths were dissected from each fish and aged whole by two independent readers. Whole otoliths with ages over two years, and any for which there was disagreement between the readers, were sectioned and aged by the same two readers. Any otoliths for which the first two readers disagreed were aged by a third reader; if the age determined by the third reader agreed with the age determined by one of the first two readers, that age was assigned to the fish. If all three readers disagreed, they aged the otolith together to reach consensus. Sex of mature fish was determined through gonad analysis and was included as a potential confounding factor in our analyses.

### 2.3. Statistical Analyses

Although fish were targeted in size classes to ensure that a wide range of sizes were collected, analyses were conducted using data from all fish collected. To determine significant differences in tissue mercury among species using all data we used a one-way ANOVA (analysis of variance). We also used one-way ANOVA to test for significant differences in tissue mercury, trophic position, fish length, and fish age among systems for each species; a Bonferroni correction was applied to adjust for multiple comparisons. 

We determined the relationship between fish tissue Hg and body length for each species in each lake using least squares linear regression, and used those relationships to estimate the lengths at which fish in each lake reached NC (0.4 ppm), USEPA (0.3 ppm), and USFDA (1.0 ppm) fish tissue Hg screening or action levels, above which consumption risk is elevated. We related these higher risk sizes to typical fishery length limits (described previously) for each species. 

We estimated the concentration of Hg at the base of the food web and rate of Hg bioaccumulation in each lake using a method described by Jardine *et al.* [49]. Briefly, for each study lake largemouth bass and bluegill Hg concentration data were combined and the values were regressed against their trophic level (measured by nitrogen isotope analysis). The slope of the relationship was used as a measure of bioaccumulation and the intercept as an estimate of the concentration of Hg at the base of the food web in that lake [20,49,50,51]. Slopes were tested for significant differences among sites using an analysis of covariance (ANCOVA). This approach allowed us to compare the concentrations of Hg at the base of the food web and the rates of bioaccumulation among lakes. Though the values we report do not necessarily reflect exact Hg values at the base of the food web, this approach is useful for providing a relative baseline that allows consistent comparisons among systems. Mercury bioaccumulation rates measured in a similar way in other studies have ranged from 0.66 to 0.81 [20,52,53]. We excluded black crappie data from this analysis because we were unable to collect crappie in all of the lakes sampled.

To assess the relative contribution of fish age and trophic position to the fish length-fish tissue Hg relationship, we used pair-wise and partial correlation analyses. Partial correlations determine the strength of the association between two variables (e.g., fish length and fish tissue Hg) while excluding the effect of other confounding factors (e.g., fish age and trophic position). These correlation coefficients were determined for fish tissue Hg with fish length, age and trophic position for each species in each lake to determine the importance of each factor to Hg accumulation. A Bonferroni correction was applied to adjust for multiple comparisons (between tissue Hg and total length, age and trophic position) for each species in each lake (overall alpha = 0.05).

To determine whether mean fish tissue Hg obtained by the North Carolina Division of Water Quality during routine sampling could be used to predict the length at which fish would reach a screening level, we calculated mean fish tissue Hg for largemouth bass within the general length range of fish this agency collected for Hg analysis (320–385 mm; the first through the third quartile of lengths collected by NCDWQ from 1990–2006) and regressed those means against the estimated length at which largemouth bass reached each screening level in each lake.

Significance of regressions and correlation analyses were determined with ANOVA and student’s t-tests. All analyses were conducted using the SAS program JMP 8.0 (SAS Institute, Cary, NC, USA) and R version 2.15.2 (The R Foundation for Statistical Computing 2012). Fish tissue Hg concentrations were log_10_ transformed to meet assumptions of normality and equality of variance. 

## 3. Results and Discussion

### 3.1. Results

#### 3.1.1. Fish Tissue Hg

**Figure 2:**
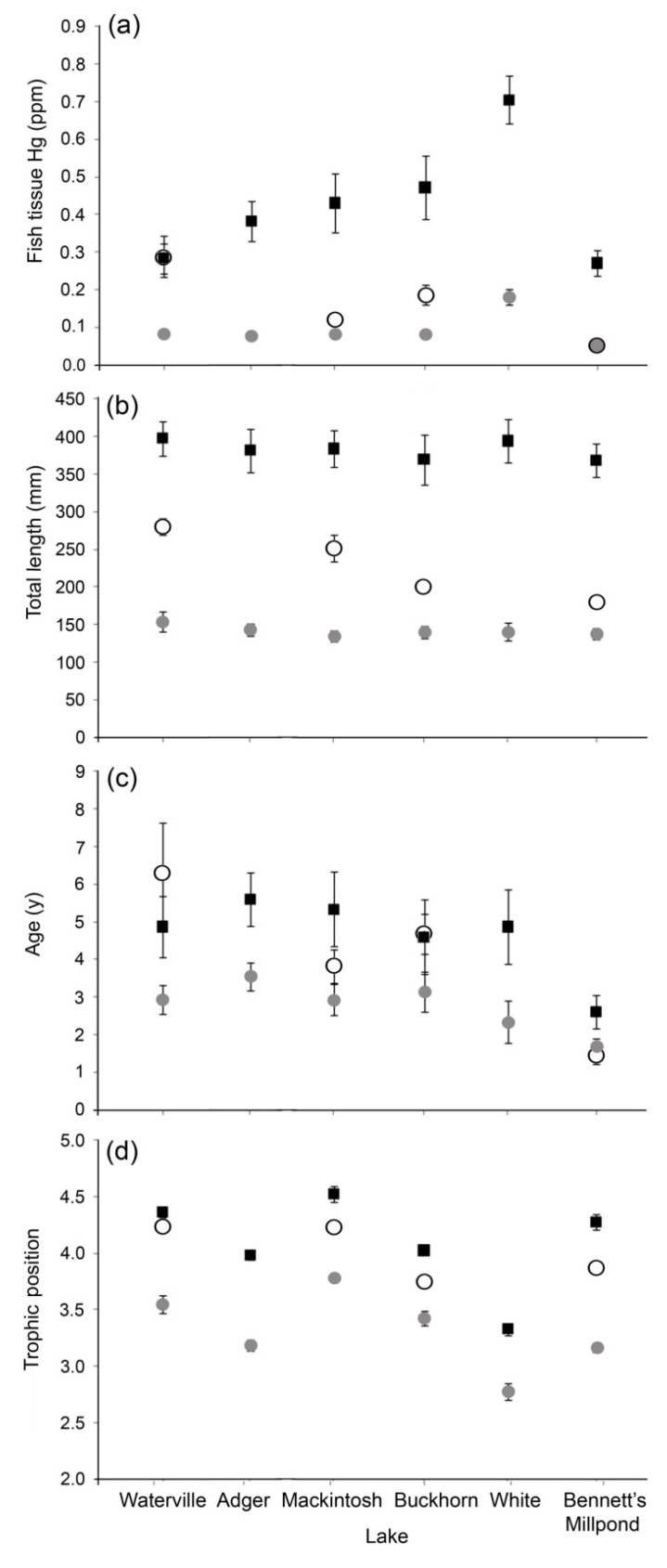
Means of (**a**) fish tissue Hg (ppm), (**b**) total length (mm), (**c**) age (years) and (**d**) trophic position (as measured by nitrogen isotopes) of bluegill (light grey circles), black crappie (open circles) and largemouth bass (dark squares) collected in four to six lakes in North Carolina, USA. Error bars indicate ± one standard error (hidden behind data symbols in some cases).

Fish tissue mercury varied among sites and species (Figure 2). Mean fish tissue Hg for largemouth bass and bluegill ranged from 0.27 to 0.70 ppm and from 0.05 to 0.18 ppm across study systems (Figure 2). The mean fish tissue Hg for black crappie ranged from 0.05 to 0.29 ppm. The partial collections of black crappie were useful in examining the relationship between fish length and Hg accumulation, but our ability to make comparisons among sites was limited. Fish tissue Hg concentrations were significantly different among species (*P* < 0.01), with largemouth bass having the greatest mean levels (0.42 ppm) followed by black crappie (0.16 ppm) and bluegill (0.09 ppm). In addition, fish tissue Hg levels were significantly different (*P* < 0.01) among lakes for each species (Figure 2(a)). White Lake had the greatest overall level of fish tissue Hg contamination whereas Bennett’s Millpond had the least. Also, mercury concentrations were not significantly different between sexes for any of our tested species (bluegill *P* = 0.56, N_female_ = 27, N_male_ = 26; black crappie *P* = 0.95, N_female_ = 19, N_male_ = 19; largemouth bass *P* = 0.10, N_female_ = 50, N_male_ = 33). 

**Figure 3:**
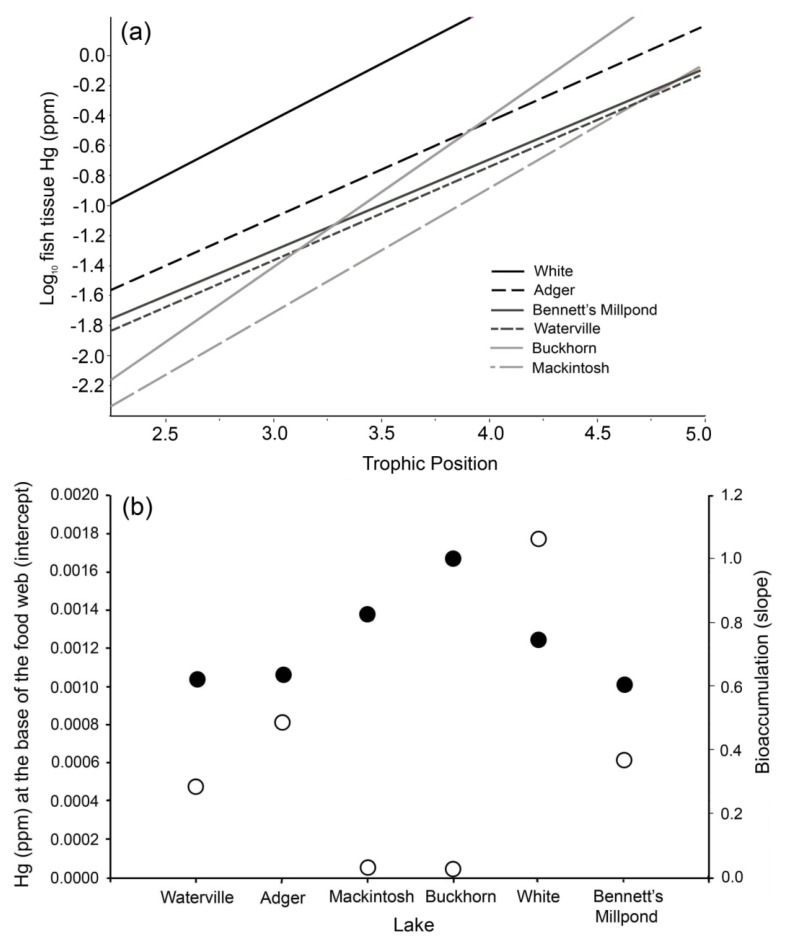
(**a**) Regression relationships for fish tissue Hg and trophic level (as measured by nitrogen isotopes) in each of six study lakes in North Carolina, USA, using largemouth bass and bluegill data. (**b**) Level of mercury at the base of the food web (y-intercept; open circles) and rate of bioaccumulation (slope; dark circles) in each study lake [20,49], determined using the regressions in (**a**).

Estimates of Hg bioaccumulation (the slope of the fish tissue Hg-trophic level relationship) ranged from 0.61 to 1.0, equivalent to a 4.0–10.0 factor increase in Hg with each trophic level, though these slopes were not significantly different (ANCOVA, *P* = 0.06, Figure 3(a)). The estimated concentration of Hg at the base of the food web (the intercept of the same relationship) in each lake ranged from 0.00003 ppm to 0.0018 ppm (Figure 3(b)). Interestingly, the two lakes with the lowest levels of Hg at the base of the food web (0.00003, 0.00005 ppm) had the greatest bioaccumulation values (1.0 and 0.83), resulting in an intermediate level of fish tissue Hg compared to other study lakes. The greatest concentration of Hg at the base of the food web was observed in White lake.

#### 3.1.2. Screening Levels and Length Limits

**Figure 4:**
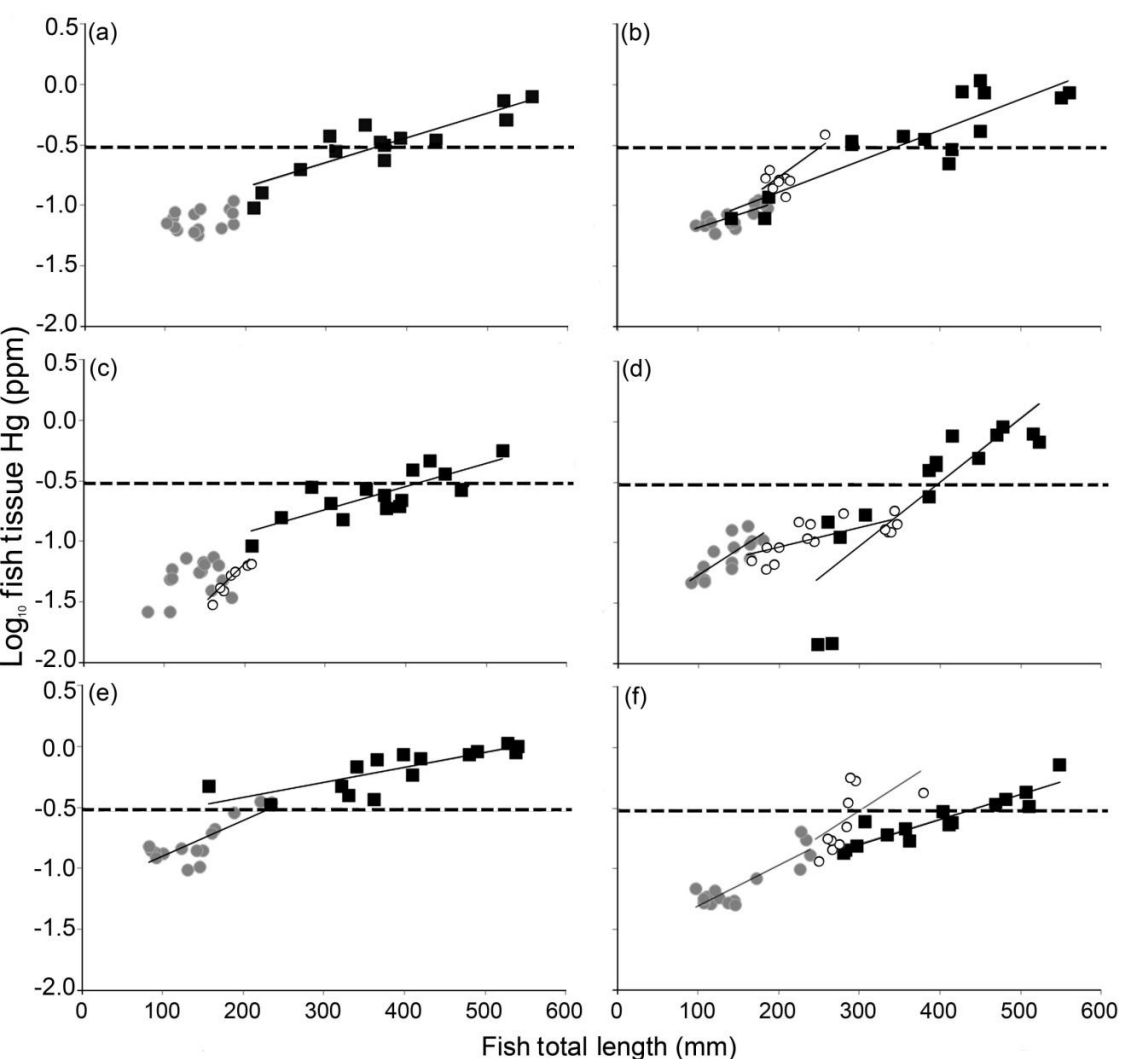
Regressions of log_10_ fish tissue Hg (ppm) on fish total length (mm) for bluegill (light grey circles), black crappie (open circles) and largemouth bass (dark squares) in lakes (**a**) Adger, (**b**) Buckhorn, (**c**) Bennett’s Millpond, (**d**) Mackintosh, (**e**) White, and (**f**) Waterville in North Carolina, USA. Solid lines represent regression equations for each species in each lake, except bluegill in Lake Adger and Bennett’s Millpond, which did not have a significant relationship (see Table 2). The horizontal dashed lines indicate the USEPA Hg screening level of 0.3 ppm.

Fish tissue Hg concentrations increased significantly with fish body length (*P* < 0.05) for every species-lake combination with the exception of bluegill in two lakes (Table 1). The regression coefficients (R^2^) ranged from 0.54 to 0.92. Significant regression equations (*P* < 0.05) were used to estimate the lengths at which each species in each lake would reach the USEPA (0.3 ppm), NC (0.4 ppm) and USFDA (1.0 ppm) screening or action levels (Table 1, Figure 4). The only bluegills to surpass the USEPA screening level were in the system with the highest levels of tissue Hg (White lake), and even there only the largest individuals (>227 mm) were of concern. Black crappie smaller than the fishery length limit (203 mm) did not surpass the USEPA, NC or USFDA screening or action levels. Legally harvestable black crappie (fish above the fishery length limit) surpassed the USEPA screening level in two lakes (Table 1); 25% surpassed the screening level in one lake (Buckhorn) and 40% in another (Waterville). Largemouth bass surpassed the USEPA screening level below the fishery length limit (355 mm) in two of the six lakes sampled (White and Buckhorn). 

In the remaining lakes, largemouth bass surpassed the USEPA screening level at or before fish reached 432 mm (17 inches), a size commonly considered by anglers as ‘desirable’ in North Carolina and elsewhere. Of the legally harvestable largemouth bass collected in each lake, 44% (Bennett’s Millpond), 50% (Waterville), 89% (Adger), 90% (Buckhorn), 90% (Mackintosh), and 100% (White) of fish were above the USEPA Hg screening level. In addition, largemouth bass surpassed the NC screening level below the fishery length limit in White Lake (Table 1), and the largest individuals (>500 mm) surpassed even the conservative USFDA action level in three lakes.

We calculated mean Hg concentrations from a subset of largemouth bass data (fish within a size range commonly caught by NCDWQ; 320–385 mm) from each sample lake and regressed them on the estimated length at which largemouth bass reached each screening or action level. The lengths at which largemouth bass reached each of the three screening or action levels decreased as mean fish tissue Hg increased (USEPA, *P* = 0.01, *R^2^* = −0.91; NC, *P* = 0.004, *R^2^* = −0.95; and USFDA, *P* = 0.03, *R^2^* = −0.83). The equations for each regression are as follows:

size fish reached USEPA screening level = 619.09 − 869.24 * (mean fish tissue Hg) (1)

size fish reached NC screening level = 654.43 − 770.61 * (mean fish tissue Hg) (2)

size fish reached USFDA action level = 767.01 − 456.47 * (mean fish tissue Hg) (3)

**Table 1:** Regression analyses of log_10_ fish tissue Hg concentrations (ppm) on fish total length (mm TL) in bluegill, black crappie and largemouth bass from lakes sampled in North Carolina, USA. Estimated lengths (mm TL) at which each species reached the USEPA (0.3 ppm), NC (0.4 ppm) and USFDA (1.0ppm) fish tissue Hg screening or action levels are also listed. Values with a greater than (>) symbol exceed the maximum size of the species found in North Carolina [35,54]. Bold text indicates significant relationships (*P* < 0.05).

Species	Lake	*P*-value	*R^2^*	Equation	Estimated length at Hg levels for		
					EPA (0.3 ppm)	NC (0.4 ppm)	FDA (1.0 ppm)
Bluegill	Adger	0.20	0.12	log_10_(Hg) = −1.262 + 0.000975 * TL	>300	>300	>300
	Bennett’s Millpond	0.20	0.12	log_10_(Hg) = −1.537 + 0.00172 * TL	>300	>300	>300
	Buckhorn	<0.01	0.56	log_10_(Hg) = −1.389 + 0.00211* TL	>300	>300	>300
	Mackintosh	<0.01	0.61	log_10_(Hg) = −1.687 + 0.00424 * TL	274.9 *	>300	>300
	Waterville	<0.01	0.76	log_10_(Hg) = −1.639 + 0.00335 * TL	>300	>300	>300
	White	<0.01	0.65	log_10_(Hg) = −1.196 + 0.00296 * TL	227.3	269.5 *	>300
Black Crappie	Adger	---	---	---	---	---	---
	Bennett’s Millpond	<0.01	0.92	log_10_(Hg) = −2.481 + 0.00651 * TL	300.9	320.1	381.3 *
	Buckhorn	0.02	0.55	log_10_(Hg) = −1.744 + 0.00489 * TL	249.8	275.3	356.7
	Mackintosh	<0.01	0.54	log_10_(Hg) = −1.357 + 0.00162 * TL	>500	>500	>500
	Waterville	0.05	0.39	log_10_(Hg) = −1.816 + 0.00431 * TL	299.9	328.9	421.3 *
	White	---	---	---	---	---	---
Largemouth Bass	Adger	<0.01	0.79	log_10_(Hg) = −1.258 + 0.00204 * TL	361.1	422.5	618.0 *
	Bennett’s Millpond	<0.01	0.64	log_10_(Hg) = −1.315 + 0.00191 * TL	414.1	479.4	687.5 *
	Buckhorn	<0.01	0.77	log_10_(Hg) = −1.396 + 0.00255 * TL	341.8	390.8	546.6
	Mackintosh	<0.01	0.73	log_10_(Hg) = −2.619 + 0.00530 * TL	395.2	418.7	493.8
	Waterville	<0.01	0.84	log_10_(Hg) = −1.412 + 0.00206 * TL	432.1	492.8	686.3 *
	White	<0.01	0.64	log_10_(Hg) = −0.659 + 0.00121 * TL	112.1	215.1	543.4

* Predicted beyond range of data (range of length data for bluegill 79–239 mm, black crappie 155–376 mm, largemouth bass 140–560 mm) but within achievable size range of the species.

##### 3.1.3. Fish Length, Age and Trophic Position

As expected (due to our length-based sampling strategy), length was not significantly different among study lakes for largemouth bass (*P* = 0.96) or bluegill (*P* = 0.81, Figure 2(b)), allowing us to compare our results among sites. However, because we were unable to collect all of the targeted black crappie, there were significant differences (*P* < 0.01) in lengths among lakes for this species. Ages of bluegill and largemouth bass were not significantly different (*P*_BG_ = 0.06, *P*_LMB_ = 0.17) among lakes, with one exception; for both species, fish collected from Bennett’s Millpond were significantly younger (*P* < 0.03) than those from other study lakes (Figure 2(c)). In addition, collected bluegill (mean age = 2.8 years) were significantly (*P* < 0.01) younger than largemouth bass (mean age = 4.6 years) overall. Trophic position, as measured by nitrogen isotopes, differed significantly among species (*P* < 0.01). Largemouth bass consistently occupied the highest trophic position, followed by black crappie and then bluegill (Figure 2(d)). However, the trophic position of each species differed among lakes. For instance, the mean trophic positions of largemouth bass and bluegill were highest in Mackintosh Lake and lowest in White Lake (Figure 2(d)).

The importance of fish length, age and trophic position to Hg accumulation varied among lakes and species (Table 2). For instance, bluegill showed a strong, significant pair-wise relationship (*r* = 0.75 to 0.87; Table 2) between fish tissue Hg and body length in four of the six lakes sampled. However, partial correlation analysis revealed weaker, insignificant relationships (Table 2), demonstrating that when fish age and trophic position were held constant, the relationship between fish length and fish tissue Hg was not as strong. However, for half of the sites from which largemouth bass and black crappie were collected, tissue Hg increased strongly with length even after the effects of the confounding factors (fish age and trophic position) were removed (Table 2).

**Table 2:** Pair-wise and partial correlations of log_10_ fish tissue Hg (ppm) with fish total length (mm TL), age (years) and trophic position (as measured by nitrogen isotopes) for bluegill, black crappie and largemouth bass collected in six lakes in North Carolina, USA. Partial correlations indicate correlations between fish tissue Hg and each factor individually while removing the effects of the other two factors. Bold text indicates significant relationships (*P* < 0.05, adjusted with a Bonferroni correction to *P* < 0.008).

Species	Lake	Hg and TL		Hg and age		Hg and trophic position	
		*r*	partial *r*	*r*	partial *r*	*r*	partial *r*
Bluegill	Adger	0.35	−0.31	0.51	0.49	−0.08	−0.12
	Bennett’s Millpond	0.35	0.07	0.16	0.01	0.55	0.47
	Buckhorn	**0.75**	−0.08	**0.75**	0.20	**0.81**	0.47
	Mackintosh	**0.78**	−0.36	**0.86**	**0.65**	−0.01	−0.10
	Waterville	**0.87**	0.46	**0.86**	−0.0034	**0.92**	**0.78**
	White	**0.80**	0.24	**0.84**	0.49	0.19	−0.15
Black Crappie	Adger	---	---	---	---	---	---
	Bennett’s Millpond	**0.96**	**0.98**	0.58	0.76	0.55	0.73
	Buckhorn	0.74	0.35	**0.90**	**0.81**	0.07	0.43
	Mackintosh	**0.73**	**0.68**	0.61	0.53	0.04	−0.0061
	Waterville	0.63	0.41	**0.89**	**0.82**	0.33	−0.19
	White	---	---	---	---	---	---
Largemouth Bass	Adger	**0.89**	**0.59**	**0.88**	0.40	−0.39	−0.17
	Bennett’s Millpond	**0.80**	0.30	**0.85**	0.45	**0.72**	0.33
	Buckhorn	**0.88**	**0.72**	**0.84**	0.58	**0.85**	**0.70**
	Mackintosh	**0.86**	**0.62**	**0.69**	−0.09	**0.73**	−0.21
	Waterville	**0.92**	0.43	**0.91**	0.41	0.27	−0.17
	White	**0.80**	0.24	**0.82**	0.40	**0.69**	0.12

### 3.2. Discussion

#### 3.2.1. Length Thresholds for Increased Risk

The lengths at which each species reached the USEPA, NC and USFDA screening or action levels varied by lake, and in some cases, fell below the fishery length limits for harvest. Different Hg screening or action levels from state, federal and international agencies make it confusing for consumers to understand the risk they face when consuming fish [55,56,57]. The USEPA screening level of 0.3 ppm is the most conservative. This screening level is based on a chronic oral reference dose of 0.1 μg/kg/day [55,58,59], which was determined to be scientifically justifiable by the National Research Council [60], and was based on neuro-developmental effects associated with in utero exposure to MeHg. 

The underlying assumption is that such values are protective for all individuals (including pregnant women and children), for two 6-ounce fish meals per week over a lifetime. The USFDA action level is 1.0 ppm, however, this is intended as a regulatory action level, used as the concentration at which the USFDA can take legal action to remove products from the marketplace [56]. Here, we focus much of our discussion on the USEPA screening level because it is protective for all potential consumers of fish over a lifetime, it pertains to sport-caught fish, and it is widely used throughout the USA [60,61,62]. Largemouth bass exceeded the USEPA screening level in all lakes prior to reaching 432 mm, suggesting all large bass in our study lakes would likely be considered a higher risk, regardless of the level of system contamination. In addition, larger black crappie also exceeded the USEPA screening level, though not at sizes below the fishery harvest size limit. In North Carolina and many other USA states there is no fishery length limit for bluegill. Our results suggest that the latter poses no major problem from a risk management standpoint in terms of Hg consumption; even in systems with high Hg methylation rates, we found bluegill generally safe to consume throughout their length range. The relatively strong relationship between fish tissue Hg and length in our investigation corroborates other studies [9,10] that have indicated that length within a species is a reliable predictor of the hazard of increased Hg exposure for fish consumers. 

The notion that consumption advisories be size-based for individual species has been previously suggested and implemented [10,63,64], and our results are consistent with that recommendation. Indeed, Burger and Gochfeld [10] suggest that fish size, for an individual species, may be the best indicator of consumptive risk for the public. However, our study extends those previous investigations by quantifying differences in Hg contamination among systems and determining the resultant effects on the sizes at which fish became higher risk. Taken together, this suggests that the most robust risk communication strategy would, where feasible, include water body specific information in addition to the species- and size-based recommendations. While this strategy has been implemented in a few states, many of these advisories seem inconsistent with fishery regulations that limit legal harvest to only those fish under advisory [65,66]. 

Although it is difficult to obtain system specific information, particularly because variables that affect Hg accumulation can change over time (e.g., food web structure, growth rates, water chemistry), many fishery and human health agencies regularly monitor fish populations and tissue Hg levels to optimize angling opportunities and protect human health. These data could be used together to inform anglers of health risks from fish consumption and allow for more harvest opportunities for sizes, species and systems where Hg is less of a concern. Our results also suggest that it may be feasible for state agencies to obtain reasonable estimates of the size at which a particular species will exceed a screening level using size-limited monitoring data. While the relationships we report can be used in North Carolina, other state agencies can establish similar predictive relationships and estimate higher risk sizes without analyzing tissue Hg from a wide size range of individuals.

#### 3.2.2. Modifying Regulations to Reduce Risk

Fish are an excellent source of lean protein and omega-3 fatty acids, which reduce heart disease and increase brain development and function [67,68,69,70]. However, high levels of mercury exposure can lead to neuro-developmental problems, lower cognition in children and higher incidence of heart disease in adults [71,72]. Fish advisories are issued to protect consumers from these adverse effects, although Karouna-Renier *et al.* [73] found that many anglers and groups sensitive to Hg were unaware of fish advisories and that this lack of knowledge resulted in greater Hg exposure. 

Additionally, though some states and regions have adopted species- and size-based fish consumption advisories, many of these same places have fishery length regulations that legally limit harvest, and thus consumption, to sizes of fish under advisory [65,66]. Fishery length limits that incorporate fish advisory and public health goals could increase awareness of the relative risk of consuming larger, higher-Hg fish while simultaneously reducing the risk of high Hg exposure to anglers and their families [74]. We suggest that public health and fishery management agencies work together to increase awareness and reduce health risks associated with consuming high-Hg fish, particularly in systems where fish exceed the Hg screening level prior to reaching the fishery length limit.

One possible modification to current regulations could be a slot length limit or maximum length limit that prevents anglers from harvesting the largest and most contaminated fish within a system; another is to limit the number of large, highly contaminated fish that can be taken by an angler [75]. Our results suggest that the lengths chosen for these limits would need to be species- and system-specific. This approach would help public policy makers balance both natural resource and human health goals. Our study also demonstrated that largemouth bass in North Carolina, and possibly in the greater southeastern United States, may require a maximum length limit for safe consumption, particularly in more contaminated systems. Alternatively, Burger and Gochfeld [10] suggested using the percent of harvestable fish (those above the fishery length limit) captured that surpassed the fish tissue Hg screening level (in lieu of mean Hg level) to inform pregnant women and children of the probability that a single meal will be higher risk. We reported these percentages here and showed that there is a 90% chance that a harvestable sized largemouth bass from two of our study lakes will exceed the USEPA screening level and a 100% chance in another. This method would take into account potentially harmful spikes in Hg from a single meal that the screening levels (based on lifetime consumption) do not.

Here we provide concrete evidence of the necessity for more communication between fishery managers and public health officials so that the message sent to anglers is more consistent. We believe that fishery regulations that take contaminant information into consideration could provide an intermediate step more protective than just an advisory, but short of an outright ban on harvest (which is rarely necessary). Data collected and expertise from both fishery and public health agencies could be used together to better inform anglers of health risks from fish consumption and allow for more harvest opportunities for sizes and species of fish in systems where Hg is less of a concern. 

#### 3.2.3. Differences among Systems

Our results demonstrate that differences in Hg contamination and bioaccumulation can affect the relationship between fish tissue Hg and fish length. Other studies have noted that the mercury-length relationship varied among systems, locales and regions, further demonstrating the need for system specific information in risk communication [76,77]. Our isotopic analysis confirmed that trophic level is a good predictor of fish tissue Hg among species [20] and that fish tissue Hg, as well as trophic level of each species, varies from system to system. The greatest tissue Hg contamination for bluegill and largemouth bass was found in White Lake, a clear, spring-fed lake in the coastal plain with a very low pH (4.64), despite the lower trophic positions of both species there than in any of the other study lakes. This result is consistent with the observation that systems with low pH located in the mid-Atlantic coastal plain typically have elevated levels of fish tissue Hg, likely due to higher net rates of Hg methylation [30,78,79]. This would further suggest that a relatively high baseline level of Hg is driving high concentrations of fish tissue Hg in White Lake, a result supported by our estimate of Hg at the base of the food web (which was greater in White Lake than any other study system).

Interestingly, the lowest fish tissue Hg concentrations were found in Bennett’s Millpond, another site in the mid-Atlantic coastal plain. Given the similar abiotic conditions, we would have expected this system to be similar to White Lake, with relatively high levels of Hg contamination [30]. Estimated Hg concentration at the base of the food web and bioaccumulation rate were, however, intermediate at this site compared to the other study systems. A likely explanation for this result is that fish were much younger in this system than in the other study lakes. The young age of fish in Bennett’s Millpond could be a function of relatively high fishing pressure (North Carolina Wildlife Resource Commission, Jeremy McCargo, personal communication) that removes the largest and oldest individuals, or fish populations may still be recovering from a major fish kill event associated with Hurricane Isabel in 2003, seven years prior to sampling for this project. In either case, younger fish in this system would not have had as much time to accumulate Hg as fish of similar sizes in the other study lakes.

Others have found similar Hg bioaccumulation rates in aquatic systems (e.g., 0.66–0.81) [20,53,80] and attributed differences in fish tissue Hg concentrations to differences in Hg at the base of the food web, presumably driven by net methylation rates and Hg inputs (e.g., deposition) [20,50,81]. Our results suggest that while estimated levels of Hg at the base of the food-web are important, bioaccumulation rates play an important part as well. For instance, two lakes (Mackintosh and Buckhorn) in the Piedmont region of North Carolina exhibited relatively low levels of Hg at the base of the food web (as expected based on our previous work) [30], but relatively high bioaccumulation rates in these two lakes caused fish tissue Hg to reach higher than expected levels. As a result, these lakes were intermediate in fish tissue Hg contamination. In lakes where largemouth bass grow less efficiently, those fish could accumulate more Hg without changing trophic position, increasing the slope of the fish tissue Hg and trophic position relationship, and thus the estimate of the rate of bioaccumulation. These results suggest that, in addition to understanding factors that influence baseline Hg contamination (e.g., net methylation), efforts to estimate Hg levels in fish may need to consider bioaccumulation rates and fish growth as well [76].

Though limited to six study systems, our investigation spanned a range of ecoregions common on the eastern seaboard of the continental US, [82,83,84], and significantly broadens our understanding of the southeastern region, an area noted for having exceptionally high levels of Hg contamination [27,83,84]. On a broader scale, this study explicitly links fishery harvest restrictions with contaminant dynamics and public health advisories, providing a template for similar research in systems throughout North America.

#### 3.2.4. Fish Length, Age and Trophic Position

Though the relationship between fish length and tissue Hg levels is well established [8,10,16,17]; many previous investigations have attributed this relationship to increases in fish age or trophic level. Our results showed that while fish age and trophic level are important determinants of fish tissue Hg, fish length can also be important to the accumulation of fish tissue Hg, independent of age and trophic level. Presumably, this relationship is due to decreased growth efficiency, becasue larger fish simply need more food for the maintenance of a larger body [14]. This increase in tissue Hg and food consumption would be unrelated to trophic position because food items would not necessarily be a higher trophic level. In addition, larger fish may have higher metabolic activity costs associated with reproductive behavior, which would also require an increased rate of prey consumption and thus Hg accumulation. 

## 4. Conclusions

Robust understanding of Hg dynamics and relative risk to human health, particularly in the context of fishery harvest regulations, requires an understanding of body length-fish tissue Hg dynamics in important sport species. Our research addressed this issue by directly assessing the relationship between fish length and tissue Hg across systems spanning a range of ecosystems and Hg contamination, for three common and widely harvested sport species. In many of our study systems, current fishery regulations restrict harvest to lengths of fish that likely have elevated Hg levels, potentially posing risk to consumers. In some systems it may be feasible to implement a maximum length limit restricting harvest to smaller fish to reduce risk to consumers, while enhancing catch-and-release opportunities for larger fish. We also suggest that the public should be informed about length-based, species- and system-specific health risks to make informed decisions about which fish to eat. For example, modifying the message to reduce risk by encouraging people to eat the fish that are safe for them is perhaps a better public health strategy than focusing on advisories of what not to eat. This paradigm shift to “safe eating guidelines” has been recommended by the USEPA. To accomplish this goal we recommend that state fisheries agencies work with public health officials whenever possible to develop fish harvest regulations that both meet fisheries management goals and reduce consumption risk. 
